# Non-pharmacological interventions to promote the sleep of patients after cardiac surgery: a systematic review ^1^

**DOI:** 10.1590/1518-8345.1917.2926

**Published:** 2017-09-12

**Authors:** Fernanda de Souza Machado, Regina Claudia da Silva Souza, Vanessa Brito Poveda, Ana Lucia Siqueira Costa

**Affiliations:** 2 Master’s student, Escola de Enfermagem, Universidade de São Paulo, São Paulo, SP, Brazil. RN, Hospital Sírio Libanês, São Paulo, SP, Brazil.; 3 MSc, RN, Hospital Sírio Libanês, São Paulo, SP, Brazil; 4 PhD, Professor, Escola de Enfermagem, Universidade de São Paulo, São Paulo, SP, Brazil.

**Keywords:** Nursing, Sleep, Thoracic Surgey, Review, Complementary Therapies

## Abstract

**Objective:**

to analyze evidence available in the literature concerning non-pharmacological interventions that are effective to treat altered sleep patterns among patients who underwent cardiac surgery.

**Method:**

systematic review conducted in the National Library of Medicine-National Institutes of Health, Cochrane Central Register of Controlled Trials, Latin American and Caribbean Health Sciences Literature, Scopus, Embase, Cumulative Index to Nursing and Allied Health Literature and PsycINFO databases, and also grey literature.

**Results:**

ten controlled, randomized clinical trials were included in this review. Non-pharmacological interventions were grouped into three main categories, namely: relaxation techniques, devices or equipment to minimize sleep interruptions and/or induce sleep, and educational strategies. Significant improvement was found in the scores assessing sleep quality among studies testing interventions such as earplugs, sleeping masks, muscle relaxation, posture and relaxation training, white noise, and educational strategies. In regard to the studies’ methodological quality, high quality studies as established by Jadad scoring were not found.

**Conclusion:**

significant improvement was found among the scores assessing sleep in the studies testing interventions such as earplugs, sleeping masks, muscle relaxation, posture and relaxation training, white noise and music, and educational strategies.

## Introduction

Sleep is described as a multidimensional, biobehavioral phenomenon with objective and subjective sensations^1^, intended for physical and mental restoration.

Altered sleep patterns is a common problem among cardiac patients^2^ and is one of the most frequently reported symptoms by patients in the postoperative period of cardiac surgery^3^. The sleep pattern of these patients is changed over the recovery period^2^ and is characterized by shorter periods, frequent awakenings and a perception of poor quality^2^ ^,^ ^4^. As a consequence of fragmented sleep, patients experience increased daytime sleepiness, fatigue and irritability, which may reduce their motivation to attend reabilitation therapy, prolong the recovery period, and increase the length of hospitalizations^5^ ^-^ ^6^.

Sleep deprivation may occur due to poor quality or a small amount of sleep hours, or be due to an imbalanced circadian rhythm^7^. In an acute situation, the quality, continuity and depth of sleep are changed by multiple factors, such as age, gender, health status, treatments, environment, pain, fatigue, psychological disorders, or prior sleep disorders^2^ ^,^ ^8^.

In this sense, it is important to understand the potential multifactorial causes of sleep disruption to develop effective therapeutic strategies, including the management of symptoms, such as pain and nausea, and environmental control, such as decreasing noise and light^2^ ^,^ ^4^.

Various techniques have been tested to promote sleep among hospitalized patients^9^. Studies suggest that a combination of interventions able to actively reduce anxiety and pain, as well as controlling for environmental factors, is efficacious in decreasing the incidence of sleep disorders^9^ ^-^ ^10^.

Therefore, seeking to contribute to greater quality of care, this study’s objective is to analyze evidence available in the literature concerning effective non-pharmacological interventions to treat altered sleep patterns among patients who have undergone cardiac surgery.

## Method

This is a systematic literature review. Systematic reviews are essential to establishing evidence-based practice, as they are a resource through which studies’ results are collected, categorized, assessed and synthesized^11^. The guidelines provided by the Cochrane Handbook concerning the development of systematic reviews were followed^12^.

The guiding question was developed using the PICO strategy^13^, where “P” (patient) refers to patients who underwent cardiac surgery, “I” (intervention) refers to non-pharmacological intervention to treat altered sleep patterns, “C” (control) refers to regular care, and “O” (outcome) refers to improved sleep patterns. Therefore, the guiding question was: what is the evidence available in the literature regarding effective non-pharmacological interventions to treat altered sleep patterns among patients who underwent cardiac surgery?

**Figure 1:**
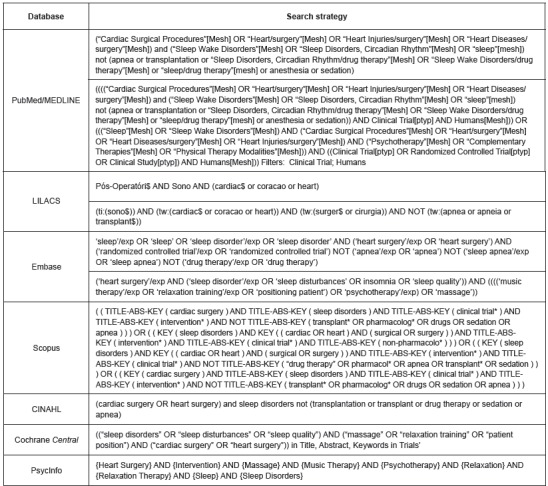
Search strategy used in the databases

The study was conducted between June and July 2016 through a search conducted in the following databases: National Library of Medicine-National Institutes of Health (PubMed/MEDLINE), Cochrane Central Register of Controlled Trials (Cochrane Central), Latin American and Caribbean Health Sciences Literature (LILACS), Scopus, Embase, Cumulative Index to Nursing and Allied Health Literature (CINAHL) and PsycINFO. A combination of controlled and uncontrolled descriptors was used to maximize the search and identify available evidence. The controlled descriptors were selected using the Medical Subject Headings Section (MeSH), Descriptors in Health Science (DeCs), Biomedical Research (Emtree) and CINAHL Headings (Figure 1).

Primary studies with designs characterized by strong evidence were included, i.e., randomized controlled clinical trials classified as providing evidence of level 2, according to Melnyk and Fineout-Overholt^14^. The studies included addressed individuals aged 18 years old or older, regardless of sex, ethnicity, or comorbidities, presenting an altered sleep pattern after having undergone myocardial revascularization surgery, or corrective surgery for valve repair or congenital heart disease, and/or aortic surgery. Studies that measure sleep patterns regardless of postsurgical phase (conducted in the intensive care unit, infirmary, or at home), written in English, Portuguese or Spanish, were also considered. No time limits were established in regard to date of publication. Studies addressing patients undergoing clinical treatment and/or percutaneous treatment of heart diseases were excluded. Ultimately, 231 studies were identified using this search strategy.

A search was also conducted in the following databases: ProQuest Dissertations and Theses, Digital Library of Theses Dissertations of the University of São Paulo, Evidence-Informed Policy Network (EVIPNet), Observatory of Intellectual Production of Medical School at the University of São Paulo, Brazilian Registry of Clinical Trials and ClinicalTrials.gov. This strategy resulted in 49 studies.

Hence, a total of 280 primary studies were initially found, while those that appeared more than once were identified to establish a final selection of papers.

In order to ensure the quality of this stage and avoid selection bias, at least two independent reviewers checked all the studies. The decision whether studies should be included in the review or not was based on the papers’ titles and abstracts. Disagreement between the two reviewers was resolved with the participation of a third reviewer. The full texts of the selected papers were assessed to ensure inclusion criteria were met. Among the reasons primary studies were excluded were: the method did not include randomized clinical trials; did not measure quality of sleep; lack of non-pharmacological intervention to promote sleep, or patients other than those in the postoperative of cardiac surgery were addressed. 

Two independent reviewers read the titles and abstracts of 25 studies, from which the full papers of 13 were selected. Disagreements between the reviewers were discussed and a third reviewer was consulted until consensus was reached. The final sample was composed of ten studies addressing the efficacy of non-pharmacological interventions among patients presenting an altered sleep pattern after cardiac surgery. Data were extracted from the ten primary papers using an instrument developed by Ursi (2005)^15^. The scoring parameters established by Jadad et al.(1996)^16^ ^)^ was used to assess the methodological quality of the randomized clinical trials.

## Results

**Figure 2:**
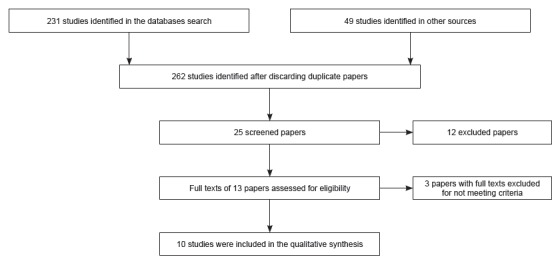
Flowchart used in the studies selection

Most studies (80%) tested interventions that promote relaxation, and, consequently, improve quality of sleep. Different methods, however, were used to achieve this objective. Figure 2 describes the non-pharmacological interventions assessed in the primary studies and their respective results concerning quality of sleep and methodology quality classification.

**Figure 3:**
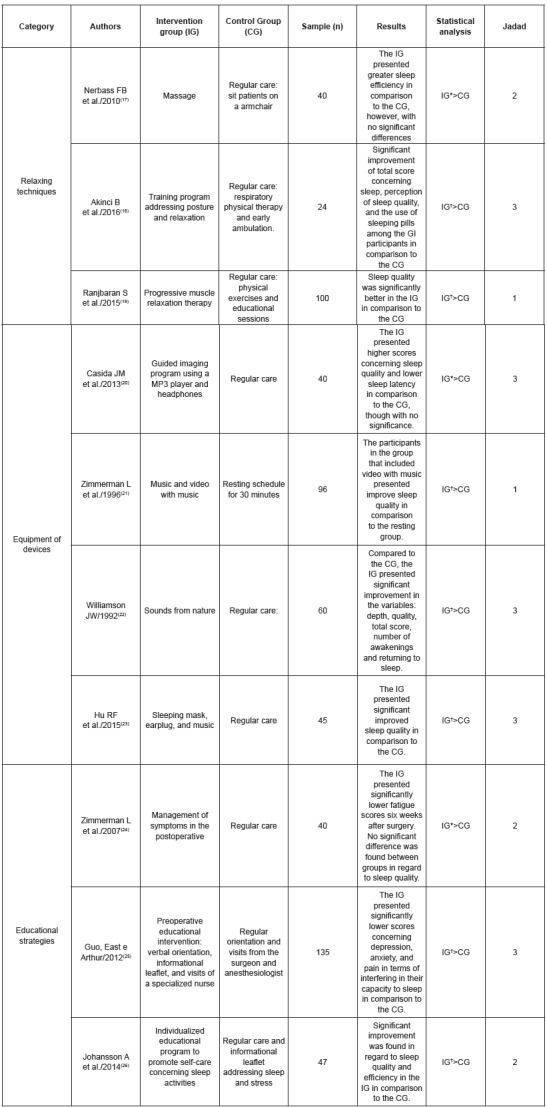
List of the selected papers, syntheses of clinical trials and their respective Jadad scores

In regard to year of publication, the studies are distributed between 1992 and 2016, though most (six) of the papers were published in the last five years. These data reveal that altered sleep pattern is a more recent topic of interest. Among the countries of origin, the United States stands out with four studies. Brazilian researchers seldom research the topic, as only one paper was authored by Brazilian authors, revealing a gap of studies in the Brazilian context.

The studies were assigned to three general categories according to the type of intervention, namely: relaxation techniques, devices or equipment to minimize sleep disruption and/or induce sleep, and educational strategies. Even though the division between categories is clear, each study fits more than one category, hence, the most marked characteristic of each study was chosen to categorize them (Figure 2).

Four^20^ ^,^ ^22^ ^-^ ^23^ of the ten primary studies included in this review tested devices that minimize sleep disruptions and/or induce sleep. The authors of three clinical trials^17^ ^-^ ^19^ investigated the efficacy of relaxation techniques and three studies^24^ ^-^ ^26^ assessed the effectiveness of educational strategies (Figure 2).

Three^20^ ^,^ ^22^ ^-^ ^23^ ^)^ of the studies categorized as concerning devices presented moderate methodological quality according to Jadad scoring. The devices used to promote sleep in these studies included: sleeping masks, earplugs, guided imagery program, and headphones.

In this review, relaxation techniques include massage, progressive muscle relaxation, and posture and relaxation training. The studies^18^ ^-^ ^19^ report significant improvement of sleep quality among those who received the different interventions. Nonetheless, even though significant improvement was reported, a need for greater methodological rigor in the studies’ development was verified. When assessing the studies using Jadad scoring, we verified that the authors of one study^19^ do not report why participants withdrew or were removed from the study; neither did they use double-blind procedures (between examiners and examinees). The need for further studies using correct and robust designs is clear, greater reliability of analyses is to be ensured.

## Discussion

Different studies show the impact of sleep deprivation in the physiological sphere and behavior of individuals, as well as its relationship with the physical and emotional recovery of patients in critical conditions^8^. Patients fatigued due to sleep deprivation after cardiac surgery present significant worsening of scores concerning mood, emotional well-being and social function in comparison to non-fatigued patients. The conclusion the author reached is that, in order to promote the recovery of patients after a cardiac surgery, actions that can limit fatigue through appropriate interventions are necessary to promote quality nighttime sleep^27^.

Nurses should value sleep as an integral part of their practice to ensure the recovery of patients, decreasing complications, costs and the length of hospitalizations. It became apparent that nurses with greater knowledge of the topic and sleep hygiene practices influence the quality of sleep as perceived by patients, which can decrease the consequences of poor sleep^28^.

All the studies included in this review^17^ ^-^ ^26^ ^)^ reveal that non-pharmacological interventions benefit patients’ sleep. Some interventions promoted decreased sleep latency and/or disruptions, while others improved the patients’ perceptions regarding quality of sleep. Another aspect worth noting refers to studies that included different interventions. These studies presented statistically significant differences, however, lacked appropriate methodological rigor^19^ ^,^ ^21^, affecting the strength of evidence found.

Additionally, according to Jadad’s methodological classification, the design of 50% of the studies^18^ ^,^ ^20^ ^,^ ^22^ ^-^ ^23^ ^,^ ^25^ presented moderate quality, showing there is a need for further research with greater methodological rigor to improve the reliability of results. These studies include those classified in the equipment category^20^ ^,^ ^22^ ^-^ ^23^, i.e., concerning the of use devices to promote sleep, such as sleeping masks, earplugs, headphones, and guided imagery programs.

Methodologically well-designed studies contribute to improved clinical practice, improve the adherence of professionals to care protocols, decrease the incidence of errors, and facilitate the implementation of preventive measures. Despite existing guidelines aiding the development of clinical studies, there is still a considerable number of flawed studies^29^.

This aspect was also highlighted in systematic reviews^30^ ^-^ ^31^ addressing sleep-promoting non-pharmacological interventions conducted in other populations. The conclusions of the authors who conducted such reviews were that further studies are needed to ensure the best intervention to improve sleep among hospitalized patients. Additionally, they highlight that heterogeneity is found regarding the populations, types of intervention, methods through which sleep patterns are measured, and duration of follow-up periods, in addition to the fact there are few randomized clinical trials^30^. Such factors hinder the creation of care protocols intended to improve quality of sleep from being implemented in clinical practice.

Three main categories were listed based on the results presented here, namely: studies using devices or equipment to minimize sleep disruptions and/or induce sleep, which totaled the largest number of studies^20^ ^-^ ^23^, followed by relaxation techniques^17^ ^-^ ^19^ and investigations addressing educational strategies^24^ ^-^ ^26^. Both categories presented the same amount of studies.

The category concerning devices or equipment to minimize sleep disruptions and/or induce sleep, earplugs and sleeping masks^23^ were efficient both in reducing environmental stimuli and in reducing interference in sleep patterns.

Studies addressing different populations^32^ ^-^ ^34^ report similar and statistically satisfactory results in regard to preventing a decrease in the baseline score concerning sleep quality, reducing the need for napping during the day, improving one’s perception of sleep quality, and requiring less time to fall asleep. Another study revealed that the use of earplugs alone is sufficient to improve the quality of sleep among cardiac patients, highlighting a method that is quite simple and inexpensive^35^.

The use of sound devices was tested in a paper included in this review^22^. Patients undergoing cardiac surgery listened to white noise (soothing nature sounds such as rain, waves or waterfalls) that isolated environmental stimuli and led to relaxation, contributing to falling asleep faster and maintaining sleep^22^. In this sense, there is evidence that sound interventions benefit the quality of sleep in different populations. White noise decreased the number of awakenings among healthy individuals exposed to an environment that simulated the noisy environment of an Intensive Care Unit (ICU)^36^. It is believed that white noise decreases the difference between baseline noise and peaks of noise, decreasing the reflex response of individuals in the face of intense stimuli, promoting a sound propagation effect in its transmission to the centers of central excitation, masking environmental noise and limiting one’s ability to discriminate easily detectable sounds^36^. Music reduces or controls stress and promotes comfort by attenuating neuroendocrine response with a consequent decrease in heart rate, blood pressure, respiratory rate, and improved sleep pattern^37^ ^-^ ^38^.

In the relaxation techniques category^17^ ^-^ ^19^, interventions such as massage, progressive muscle relaxation, and posture and relaxation training stood out.

Progressive muscle relaxation was beneficial in alleviating anxiety and pain, which influence sleep quality and, therefore, can also be included in care practice^39^. 

A Brazilian study included in the review, however, which investigated massage among patients in the postoperative phase of cardiac surgery, does not report significant improvement in sleep quality^17^, though another study addressing this same intervention among elderly women with heart diseases hospitalized in an ICU reports positive effects on sleep efficiency^39^.

The studies categorized as educational strategies describe educational interventions addressing general care in cardiac surgery and sleep hygiene^24^ ^-^ ^26^, reporting significant improvement in scores concerning anxiety, depression and lower interference of pain in one’s ability to sleep in comparison to those who did not receive the educational intervention^25^. In regard to sleep hygiene^26^, the intervention group reported improved sleep quality.

Patient education, an easily accessible tool of low cost and implemented by a multidisciplinary team, should be extensively encouraged in clinical practice, which also corroborates the importance of patient and family-centered individualized care, resulting in greater involvement and satisfaction. Education provided to the patient/family is currently a relevant issue, such that schools preparing health workers should include in their curricula training to qualify these professionals to educate patients^40^. 

Therefore, it is essential that nurses acquire knowledge concerning care that promotes sleep in order to actively decrease the factors that worsen patients’ quality of sleep and therefore, impact patients’ recovery, especially in regard to educational content^41^. Care that enables the implementation of various low or no cost strategies that can be implemented to achieve this objective should also be promoted.

## Conclusion

The results of this review encourage reflection on the need to produce scientific knowledge with greater methodological rigor in the field of sleep promotion during the hospitalization of surgical patients.

This review’s results indicate that studies assessing interventions, such as earplugs, sleeping masks, muscle relaxation, posture and relaxation training, white noise and educational strategies, report significant improvement in the scores assessing sleep.

These studies suggest that the non-pharmacological interventions contained in the selected studies to promote better sleep can be planned, implemented and assessed by nurses. Despite these findings, gaps in knowledge were identified so that future studies focusing on the use of non-pharmacological measures to promote sleep among patients who have undergone cardiac surgery are encouraged.
