# Erratum: Non-pharmacological interventions to promote the sleep of patients after cardiac surgery: a systematic review

**DOI:** 10.1590/1518-8345.0000.4098

**Published:** 2023-11-13

Regarding the article “Non-pharmacological interventions to promote the sleep of patients after cardiac surgery: a systematic review”, with DOI number: 10.1590/1518-8345.1917.2926, published in the Rev. Latino-Am. Enfermagem, 2017;25:e2926, page 1:

Where was written:

“ ^1^ Paper extracted from Master’s Thesis “Evidence of non-pharmacological interventions for the treatment of sleep pattern changes in patients undergoing cardiac surgery: a systematic review”, presented to Escola de Enfermagem, Universidade de São Paulo, São Paulo, SP, Brazil.” 

Now read:

“ ^1^ Paper extracted from Master’s Thesis “Evidence of non-pharmacological interventions for the treatment of sleep pattern changes in patients undergoing cardiac surgery: a systematic review”, presented to Escola de Enfermagem, Universidade de São Paulo, São Paulo, SP, Brazil. This study was financed in part by the Coordenação de Aperfeiçoamento de Pessoal de Nível Superior (CAPES) - Finance Code 001, Brazil.” 

